# Taurine Grafted Micro-Implants Improved Functions without Direct Dependency between Interleukin-6 and the Bile Acid Lithocholic Acid in Plasma

**DOI:** 10.3390/biomedicines10010111

**Published:** 2022-01-06

**Authors:** Armin Mooranian, Corina Mihaela Ionescu, Susbin Raj Wagle, Bozica Kovacevic, Daniel Walker, Melissa Jones, Jacqueline Chester, Thomas Foster, Edan Johnston, Sanja Kojic, Goran Stojanovic, Momir Mikov, Hani Al-Salami

**Affiliations:** 1 The Biotechnology and Drug Development Research Laboratory, Curtin Medical School & Curtin Health Innovation Research Institute, Curtin University, Bentley, Perth, WA 6102, Australia; A.Mooranian@curtin.edu.au (A.M.); c.ionescu@postgrad.curtin.edu.au (C.M.I.); susbinraj.wagle@postgrad.curtin.edu.au (S.R.W.); bozica.kovacevic@postgrad.curtin.edu.au (B.K.); daniel.walker1@postgrad.curtin.edu.au (D.W.); melissa.a.jones@postgrad.curtin.edu.au (M.J.); j.chester@student.curtin.edu.au (J.C.); thomas.p.foster@student.curtin.edu.au (T.F.); edan.johnston@student.curtin.edu.au (E.J.); 2 Hearing Therapeutics, Ear Science Institute Australia, Queen Elizabeth II Medical Centre, Nedlands, Perth, WA 6009, Australia; 3 Faculty of Technical Sciences, University of Novi Sad, Trg Dositeja Obradovica 6, 21000 Novi Sad, Serbia; sanja.kojic.ns@gmail.com (S.K.); sgoran@uns.ac.rs (G.S.); 4 Department of Pharmacology, Toxicology and Clinical Pharmacology, Faculty of Medicine, University of Novi Sad, Hajduk Veljkova 3, 21101 Novi Sad, Serbia; momir.mikov@mf.uns.ac.rs

**Keywords:** taurocholic acid, tissue engineering, tissue encapsulation, transplantation, surgery

## Abstract

A recent study showed an association between diabetes development and the bile acid lithocholic acid (LCA), while another study demonstrated positive biological effects of the conjugated bile acid, taurocholic acid (TCA), on pancreatic cells. Thus, this study aimed to encapsulate TCA with primary islets (graft) and study the biological effects of the graft, post-transplantation, in diabetic mice, including effects on LCA concentrations. Sixteen mature adult mice were made diabetic and randomly divided into two equal groups, control and test (transplanted encapsulated islets without or with TCA). Graft pharmaceutical features pre-transplantation, and biological effects including on LCA concentrations post-transplantation, were measured. TCA-microcapsules had an oval shape and similar size compared with the control. The treatment group survived longer, showed improved glucose and interleukin-6 concentrations, and lower LCA concentrations in plasma, large intestine, faeces, liver and spleen, compared with control. Results suggest that TCA incorporation with islets encapsulated graft exerted beneficial effects, but there was no direct and significant dependency between concentrations of interleukin-6 and LCA.

## 1. Introduction

Diabetes mellitus has been reported as a major leading cause of mortality globally, and more than 380 million patients have been diagnosed with diabetes over the last few years, with prevalence rising at an alarming rate [1]. Type 1 diabetes (TID) is an autoimmune metabolic condition in which, β-cells of the pancreas are destroyed by the immune cells, hence patients with T1D require administration of exogenous insulin to control blood glucose concentrations [2,3,4]. Although islet transplantation has shown promising results in several clinical trials, its success and wide use in clinical settings has been hindered by challenges such as immune rejection, formation of islet amyloid, and poor revascularization of transplanted graft promptly and adequately to allow for full cell recovery and survival for not only immediate but long-term graft success [5]. Inflammation and inflammatory mediators and cytokines remain major obstacles in islet delivery and transplantation, particularly in the long term. Transplanted islet grafts become damaged and eventually die in diabetic hosts due to many challenges including unwanted cell-mediated autoimmune response and upregulation of cytokine secretion such as interleukin-6, which results in graft failure, poor glycaemic control and recurrence of diabetes [6,7]. Hence, inflammation plays a major role in the failure of transplanted graft and islets’ death.

Various pro-inflammatory and anti-inflammatory cytokines have been studied in the context of islets’ survival and functions. In a study by Park et al., the role of proinflammatory cytokines was examined in islet amyloid formation and pancreatic β-cell ototoxicity [5]. The authors demonstrated that the dual role of the proinflammatory cytokine interleukin-1β is by mediating amyloid-induced protein upregulation and β-cell apoptosis as well as by inducing impaired cellular regulatory mechanisms resulting in potentiation of amyloid formation, directly affecting β-cell ability to function and produce insulin. The authors suggested that controlling the expression of interleukin-1β may provide a new pathway to preserving pancreatic β-cells and improving glycaemic control and diabetes treatment. They concluded that the proinflammatory cytokine, interleukin-1β, plays a dual role in islet survival and cellular toxicity, which has significant implications in islet transplantation and diabetes treatment. In another study, Sefina et al. explored the applications of the proinflammatory cytokine, interleukin-17A, in islet survival and functions. The authors investigated the association of this cytokine with human autoimmune disease, with a particular focus on T1D and the insulin-producing cells, pancreatic β-cells in the islet of Langerhans [8]. The authors examined the secretion of interleukin-17A in response to β-cell autoantigens, and interleukin-17A gene expression in islets, in new-onset T1D patients and those patients who died after diagnosis with T1D, as well as control. The authors found that circulating interleukin-17A(+) β-cell-specific immune-cell based proteins were strongly featured in T1D and may be used for future diagnosis of the disease. In other published studies, the proinflammatory cytokine interleukin-6 has been linked directly to islet autoantibodies expression and development and progression of T1D [9]. Accordingly, for successful islet transplantation, new robust pharmaceutical delivery systems need to be developed that can accommodate for the host inflammatory response and support islet survival and functions, to ultimately ensure graft success, particularly, in the long term.

Various transplantable or injectable pharmaceutical delivery systems have been proposed for efficient islet delivery and diabetes treatment. They include viable islet encapsulation systems with polymers and gels that can support and protect islet functions, post-transplantation. To date, long-term islet survival and functions, and complete glycaemic control remain a challenge in a widespread clinical setting. A number of studies have revealed that microencapsulation of islets improved the survival rate and blood glucose management in diabetic animal models, possibly through enhanced stability and immuno-protective effect that reserve the functionality of encapsulated islets and their short and long term insulin secretion [10].

Recent studies have explored the incorporation of human-endogenous bile acids for the delivery of either drugs or islets, for diabetes treatment [11,12,13,14,15,16,17,18,19,20,21,22,23,24,25,26,27,28,29,30]. The idea was to investigate if human-produced bile acids—notionally known to assist in food digestion and absorption—can be used as stabilising excipients and cell-protective additives, in delivering therapeutics and treating diabetes. Recent studies have shown that the incorporation of some bile acids promoted the strength and stability of microcapsules for drug and cell delivery [12,31,32,33,34,35,36,37,38,39,40,41,42,43,44,45,46,47,48,49,50,51,52]. One of the mechanisms that bile acids support cellular functions is by improving the inflammatory profile. Such mechanisms include ameliorating proinflammatory cytokines and/or increasing anti-inflammatory cytokines concentrations. In a recent study in our laboratory, we have shown that the bile acid taurocholic acid (TCA) has exerted positive biological effects on a mouse-cloned cell line of pancreatic β-cells for islet delivery, mainly through improving the inflammatory profile and reducing concentrations of interleukin-6 (IL-6) [53]. More recent studies in our laboratory have demonstrated that reducing concentrations of the secondary bile acid, lithocholic acid (LCA), improves glycaemic control and diabetes treatment [25]. Accordingly, this study aimed to investigate the application of TCA in islet transplantation and the association of treatment with concentrations of LCA, in the context of diabetes treatment.

## 2. Materials and Methods

Materials used for the fabrication of the microcapsules were purchased from Scharlab S.L (Barcelona, Spain), Sigma-Aldrich (Sydney, NSW, Australia) and Thermo Fisher (Sydney, NSW, Australia) and used as per our established methods [20,21,24,26,33,54,55,56]. Low viscosity sodium alginate was prepared as 1.2%, poly-l-ornithine was prepared as 0.13%, while the ultra-soluble gel was prepared as 1.3%. TCA was prepared as 1%. The vehicle in all mixtures was HPLC water. The mixtures were stirred for 6 h at 37 °C prior to islet encapsulation and microcapsules’ formation. Barium chloride solution was used as the bathing gel. Materials used for islet transplantation surgery were purchased from Able SCIENTIFIC (Perth, WA, Australia). Reflex7 skin closure system clips and sutures from Vilet and Ethicon were also purchased and used as per approved protocols. Blood glucose and insulin kits were purchased as Accu-Chek (Roche Diagnostics, Perth, WA, Australia) and ultrasensitive insulin kits from Mercodia (Uppsala, Sweden) and used in the pooled samples. For microcapsule imaging, platinum coating was used and supplied by the John De Laeter Research Centre (Bentley, Australia). Materials and kits used for flow cytometric analyses of inflammatory cytokines were purchased from BD Biosciences CBA technology (San Jose, CA, USA), while materials used for bile acid analyses were purchased from Merck (Melbourne, VIC, Australia), as described in our published studies [16,20,47,51,57,58]. For islet harvesting and pre-encapsulation, they were cultured using RPMI media, supplemented with 6 millimolar glucose, 10% foetal bovine serum and 1% antibiotics (Sigma-Aldrich Sydney, NSW, Australia).

### 2.1. Graft Imaging and Confocal Microscopic Analyses

For graft imaging, scanning electron microscopy, micro-CT analysis and confocal imaging were carried out on the two grafts, one with and the other without the bile acid TCA. The analyses were carried out in John De Laeter research centre, Commonwealth Scientific and Industrial Research Organisation, as well as other Curtin-based institutions (Bentley, Western Australia, Australia). Instruments used were a Zeiss Neon 40EsB (Wetzlar, Germany), Olympus IX-51 LM (Shinjuku, Tokyo, Japan), micro-CT 11.5 µm pixel (Boston, MA, USA), Oxford Instruments Aztec X-Act (Concord, MA USA) and Nikon A1 confocal system (Nikon Corporation, Tokyo Japan). For the scanning electron microscopy, the grafts were coated with platinum before imaging. The used methods were based on our well-described and published studies, and islet inclusion was as per our protocols and Curtin University procedures [16,19,23,24,26,36,37,43,45,46,55,59].

### 2.2. Graft Preparation and Surgical Transplantation

All experiments were approved by the Animal Ethics Committee at Curtin University and all experiments were performed according to the Australian Code of Practice for the care and use of animals for scientific purposes (Ethics number: AEC_2015_32).

**Figure 1:**
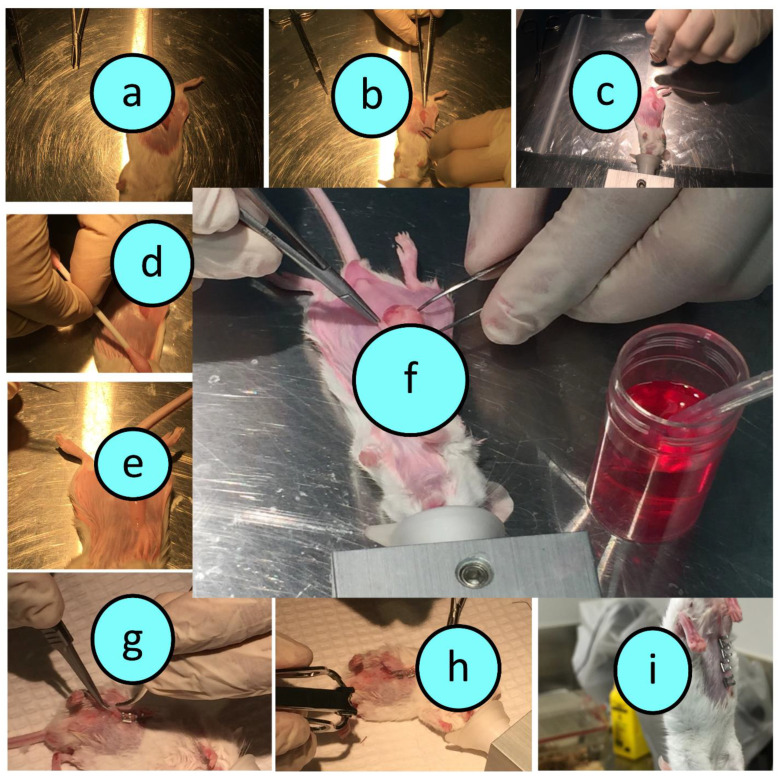
Transplantation of TCA-encapsulated islets into T1D mice (an illustration of the surgical procedure). An incision was performed (**a**–**e**), microcapsules implanted (**f**) and incision closed (**g**–**i**).

Male balb/c mice *(n* = 6*)* were purchased from Animal Resources Centre (Perth, Australia). Islets were harvested from healthy balb/c mice post euthanasia as per approved protocols using collagenase digestive enzyme. Post harvesting and digestion, islets were processed, centrifuged for 2 min and resuspended in media as per established protocols in rodent islet harvesting. Using an optical microscope, viable islets were collected (averaging 100 islets per mouse) and left in the incubator at 37 °C for 4 h prior to encapsulation and transplantation. Islet encapsulation was carried out using our ionic gelation vibrational jet technology as per our well-established protocols in pancreatic β-cell encapsulation [34]. Upon islet encapsulation, they were transplanted into the diabetic mice as shown in Figure 1a–i.

To induce diabetes, a single dose of 150 mg/kg body weight of alloxan was injected, intraperitoneally or subcutaneously, to 16 recipient mice. Mice became diabetic within 3 days of alloxan injection. Mice were provided with free access to food and water throughout the experiment. Mice were considered diabetic once their blood glucose concentrations were >16 mM and showed no insulin in plasma. The transplantation process was carried out in accordance with the surgical protocol that was documented in the animal ethics application at Curtin University. The 3Rs principle (replacement, reduction and refinement) was strictly adhered to ensure optimal animal welfare was maintained.

During the transplantation surgery, the fat pads within the visceral pelvic region were identified, isolated, stretched, inoculated with the graft and placed back surgically and aseptically. Antibiotics were applied prior to the incision, during the incision and at the end of the surgery in order to prevent any potential bacterial infections. At the end of the surgery, mice were placed in a warm specialised container in order to ensure the most comfortable conditions for recovery and ensure the most optimal surgical outcome. Soft food and easily accessible water were provided post surgery. The use of antibiotics and after-surgery care were applied to ensure optimal recovery and opportunity for optimum graft performance. Our mouse model of T1D is severely diabetic, and hence, all mice had reached significant diabetic symptoms prior to the graft transplantation surgery. Since our mouse model of T1D is acute, which ensures complete cessation of insulin production and significant and severe hyperglycaemia were achieved prior to graft transplantation. Detailed monitoring sheets were used to ensure best practice. Accordingly, the lifespan of the experiment was only a few days and no insulin was used. Mice were culled as per approved protocols to ensure animal welfare remains paramount and prevent animal suffering. The monitoring sheets included weight monitoring, infection monitoring, blood glucose measurements, and pain inspection and grading, based on the GRIMACE scale report [60,61,62] as well as other common symptoms of diabetes (Figure 1).

### 2.3. Study Design

**Figure 2:**
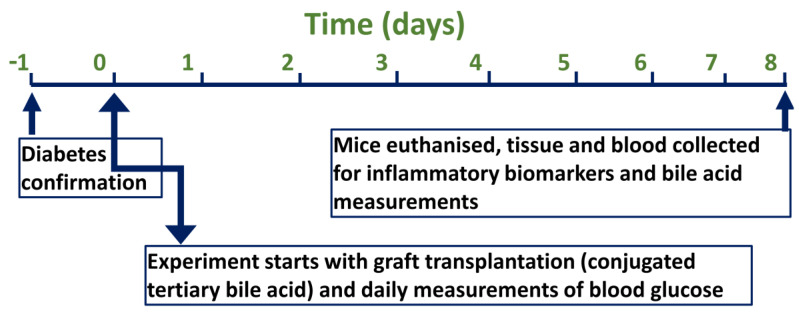
Study design and time–event series of experiments.

Figure 2 outlines the study design and time–event series of experiments for both groups, control (transplanted encapsulated islets without the bile acid TCA) and test (transplanted encapsulated islets with TCA). All experiments were performed according to the Australian Code of Practice for the care and use of animals for scientific purposes.

### 2.4. Weights Measurements, and Blood Glucose and Plasma Cytokine Analyses

In order to assess the biological performance of the transplanted grafts, blood glucose, weight measurements, survival rate and cytokine analyses were carried out for recipient mice in both groups, control and treatment. Mice weight and blood glucose were monitored as described above and based on the ethics approved by the Animal Ethics Committee at Curtin University. For diabetes confirmation, glucose and insulin levels were assessed as per our well-established methods [26,63,64]. For assessment of the inflammatory profile, concentrations of the proinflammatory cytokines interleukin-6 (IL-6), interferon-gamma (IFN-γ), tumour necrosis factor-alpha (TNF-α), interleukin 1-beta (IL-1β), interleukin-12 (IL-12), and concentrations of the anti-inflammatory cytokine interleukin-ten (IL-10) were all measured. Blood samples were collected and pooled plasma samples were used to analyse the cytokines, via the Attune Flow Cytometer (Life Technologies, Carlsbad, CA, USA). Kits for cytokine bead array (CBA) for each inflammatory cytokine were purchased and used as per manufacturer’s protocols (BD Biosciences, Franklin Lakes, NJ, USA) and based on our well-established systems [17,23,37,38,65,66].

### 2.5. Lithocholic Acid Quantification in Blood, Tissues and Faeces

Liquid chromatography-mass spectrometry (LCMS) was utilised in quantifying LCA concentrations in blood (plasma), various tissues (brain, kidney, stomach, small intestine, large intestine, liver, spleen, heart, skeletal muscles and white adipose tissues) as well as faeces. Pooled samples from mice were collected and analysed as per our well-established protocols [25]. The LCA analytical protocol was developed and established using the LCMS 2020 system (Shimadzu, Kyoto, Japan) comprised of a DGU-20A3 prominence degasser attached with SIL-20AC HT prominence autosampler (Shimadzu, Kyoto, Japan) for automated sample analyses. The quality control standards for LCA were prepared at concentrations from 1 to 1000 ng/mL including five samples within the expected range of sample concentrations with at least one to two samples lower than the expected sample concentration, in order to ensure the best accuracy in sample analysis. There was an initial run in order to determine the expected range concentration, per tissue, blood or faecal sample. The mobile phase was prepared by mixing 65% methanol and 35% water and pH tailored as appropriate, using an acid or a base, based on our established methods [16,46,51]. The prepared reagents and solvents used for sample analyses were stored at room temperature, and the remaining solutions were discarded after 1 week of preparation, if unused. LCMS sample analyses were performed with a C18 column (5 µm SPP particles, 100 mm and 2 mm internal diameter, all purchased from Phenomenex, Torrance, CA, USA). The analytical conditions used in order to do sample running included a flow rate of mobile phase at 0.25 mL per minute over the full sample run. The run samples included blank and quality control, as appropriate. The retention time was calculated based on the best elution time and based on our previously established methods with taking into account adding at least 5 min of no peak elution time to the column, ready for the next sample. The retention time for LCA was determined to be 6.5 min. The electrospray ionization probe was a built-in addition to the LCMS Shimadzu system in order to ensure a negative electrospray ionization model was obtained with mode set incorporation into the autosampling system and reached ionization mode polarity of approximately 1.5 L/min nebulization elusion gas parameter and 10 L/min gas processes to ensure consistent elusion time.

### 2.6. Statistical Analysis

Standard statistical analyses were carried out using parameter/non-parameter *t*-test or one-way ANOVA followed by ad hoc as appropriate. Data were presented as the mean plus/minus SEM or if stated otherwise. *p*-values were considered significant if *p* < 0.05 or highly significant if *p* < 0.01.

## 3. Results and Discussion

**Figure 3:**
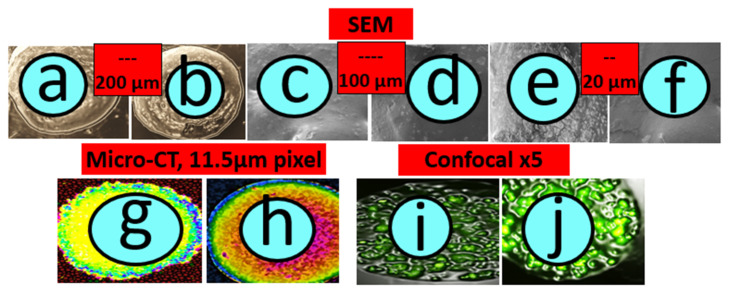
SEM (control: (**a**,**c**,**e**) and treatment: (**b**,**d**,**f**)), micro-CT (control: (**g**) and treatment: (**h**)), and confocal (control: (**i**) and treatment: (**j**)) images.

**Figure 4:**
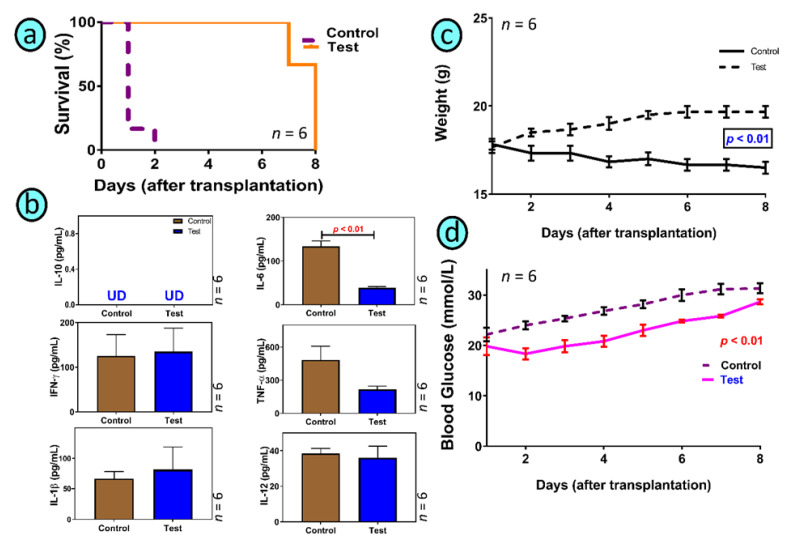
Survival (**a**), plasma cytokine concentrations for IL-10, IL-6, IFN-γ, TNF-α, IL-1β, and IL-12 (**b**), body weight (**c**) and blood glucose concentrations (**d**). Data are the mean ± SEM.

**Figure 5:**
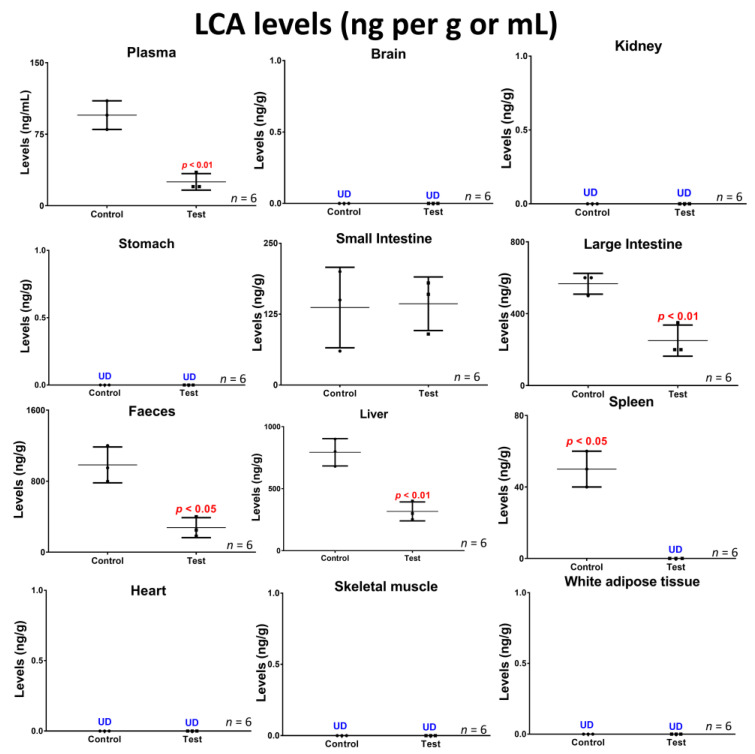
LCA concentrations in plasma, brain, kidney, stomach, small intestine, large intestine, faeces, liver, spleen, heart, skeletal muscle, and white adipose tissue in control and treatment groups. Data are the mean ± SEM.

**Figure 6:**
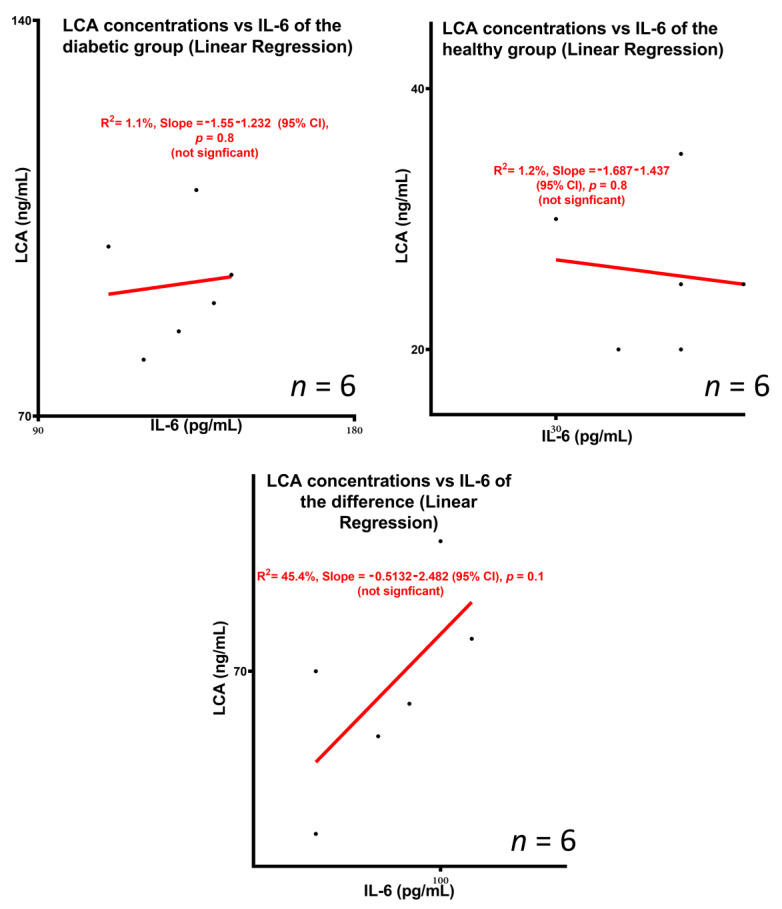
Linear regression between LCA concentrations and the proinflammatory cytokine IL-6.

Figure 3 shows the SEM images of control (a,c,e) and treatment (b,d,f), micro-CT diagrams of control (g) and treatment (h), and confocal images of control (i) and treatment (j). Figure 4 shows the survival rate (a), the pro-inflammatory and anti-inflammatory cytokines concentrations (b), mice body weight (c) and blood glucose concentrations, measured daily throughout the duration of the experiment (d). Figure 5 shows the LCA concentrations in plasma, brain, kidney, stomach, small intestine, large intestine, faeces, liver, spleen, heart, skeletal muscle and white adipose tissue, while Figure 6 shows the correlation between the concentration of LCA and the proinflammatory cytokine, IL-6.

### 3.1. Graft Imaging and Microscopic Measurements 

As shown in the SEM images, TCA-grafts presented with smoother surface characteristics compared with the control. However, there were no significant and visible changes to the shape, size or overall appearance between both grafts, which suggests that our encapsulation method is robust and consistent regardless of bile acid incorporation. The smoother surface of the TCA-graft suggests that bile acid incorporation resulted in improved surface features of the graft, reduced cracks and impediments, and unified the process of islet encapsulation, which is likely to maximise and evenly spread the number of islets encapsulated within the TCA-graft. In addition, the smoother surface may bring about improved biological effects by reducing the possibility of graft bulging and distortion. This is consistent with the literature. Several studies from other labs including de Vos P, et al. have associated graft bulging and distortion, post-transplantation, with stronger immune response, immune rejection, fibrotic growth and graft failure [1,67,68]. Others have used immune regulation agents for short- or long-term observation. As shown in the micro-CT analyses, TCA-graft treatment showed consistent and visible red dots within the graft’s layers, suggesting bile acid distribution across and throughout the graft, compared with the control. Such a cross-sectional distribution of the bile acid TCA suggests direct contact and interaction with the encapsulated islets and potential direct biological effects. As shown in the confocal images, and consisting of the SEM and micro-CT images, confocal analyses show widespread NIT-1 pancreatic β-cell distribution within the graft, visibly observed in the TCA-graft treatment image.

Several studies in our laboratory have demonstrated beneficial effects of bile acid incorporation into transplantable grafts or orally administered drug microcapsules, via smoothing and refining the surface of these grafts and microcapsules, improving microenvironment of the capsules through membrane and core stabilising effects [63,69]. It is evident from the images that the matrix as well as the encapsulating methods are robust and have enabled cellular distribution within the graft, which suggests that the encapsulating method is appropriate for islet encapsulation and bile acid incorporation did not compromise islet inclusion within the microcapsules. Accordingly, TCA incorporation is likely to exert beneficial biological and stabilising effects with smoother and more firm grafts.

Overall, improved biological effects are anticipated to encompass improved survival rate, ameliorated inflammatory profile, and better glycaemic control (Figure 4).

### 3.2. Graft Biological Effects in Diabetic Mice

As shown in the survival figure, and due to the acute diabetic state of our well-established T1D mouse model [24], control mice were culled within two days of islet transplantation, while treatment mice survived several days longer. Although control graft might have produced some insulin, and due to strict animal welfare conditions, control mice did not show sufficient improvement in terms of diabetes progress, blood glucose concentrations or signs and symptoms of the disease suggesting that the transplanted islets did not work effectively. The animal model was well established as a severe type-1 diabetic model based on our previously published studies [20,26,38,66,70,71,72]. The treatment group did better and showed improved glycaemic control and inflammatory profile as the result of TCA incorporation, although body weight remained stable. This is consistent with Figure 3 showing improved topographic features of the treatment graft as a result of TCA addition. The short survival rate is different from some studies by other laboratories that have shown longer survival and an improved glycaemic profile, post-islet transplantation. Sang-Man J et al. studied the benefits of PEGylation in early post-transplant of islets. The authors showed the ability to maintain survival and graft functions for up to a month. Their T1D rat model showed improved glycaemia although the model exhibited less severe diabetes development and diabetes symptoms compared with our mouse model [73]. In another study by Loganathan et al., and similar to a study by Sang-Man et al., the authors maintained diabetic mice for a month. The authors examined various factors affecting islet transplantation outcomes in T1D. The authors’ mouse model was nude mice, receiving human, porcine and nonhuman primate islets. The authors used a large cohort of nude mice (335) for the study and concluded that islet mass, purity, pellet size, insulin profile and the body weight of recipient mice were all factors influencing the outcome of the transplant [74]. Similarly, Stokes et al. examined hypoxia-induced factor-1α on transplanted islet survival. The authors found that decreased islet apoptosis, resulted in increased pancreatic β-cell mass post-transplantation and modulation of the hypoxia-induced factor-1α played an important role in islet inflammation, islet survival and functions and graft efficacy post-transplantation [75]. In our study, the inflammatory profiles were similar between treatment and control although IL-6 was significantly reduced in the treatment group compared with control. The reduced IL-6 concentrations suggest an improved inflammatory profile in the treatment group compared with control although none of the other measured pro- (IFN-γ, IL-1β, TNF-α, and IL-12) and anti-inflammatory (IL-10) cytokines were significantly changed. The lack of significant change on the other cytokines compared with IL-6 may be due to the short duration of the study or due to the lack of widespread anti-inflammatory effects brought about by the TCA-graft. Previous studies in our laboratory have shown anti-inflammatory effects of TCA on pancreatic β-cells, which is consistent with this study [53]. It is worth stating that in our study, the islet-loaded grafts did not elicit an immune reaction in recipient mice after transplantation, which suggests that the TCA-graft was biocompatible. Published studies on islet transplantation outcomes, carried out by different groups, have demonstrated complex, and cellular dependent mechanisms affecting islet oxygen availability [76], excipient and effects of formulation cell compatibility biomaterials [77], and the degree of immunosuppression applied during islet transplantation [78]. Others proposed that the degree of vascularisation may drastically affect the success of islet-graft success, post-transplantation [79]. In a recent study in our laboratory, the bile acid lithocholic acid (LCA) has been associated with the severity of diabetes development and progression [20]. In this study, LCA concentrations were measured in both groups of mice, control and treatment (Figure 5) and the potential association with IL-6 concentrations was assessed (Figure 6). Figure 5 showed significant alteration in LCA levels, which seem to be organ specific and suggest that diabetic control, glucose regulation and insulin release are interlinked and associated with bile acid pool, the ratio of bile acids of primary to secondary and the way tissues reserve bile acids post diabetes induction and islet transplantation. 

### 3.3. Concentrations of the Bile Acid Lithocholic Acid and Potential Association with Inflammation 

As shown in this study’s LCA profile as well as the LCA vs. IL6 linear regression analyses, TCA-graft brought about significant changes in tissue and faecal concentrations of LCA, compared with control, while no significant dependency or predictability between LCA and IL-6 profiles was found. Overall, there was a reduction in LCA concentrations in the treatment group compared with control, suggesting modulatory effects of the secondary bile acid profile brought about by TCA incorporation into the islet-graft. Such effects were likely to be due to biological effects of the TCA-graft rather than direct effects due to the presence of TCA, per se. The results showed that there was no significant dependency between the LCA profile and the IL-6 profile, which suggests that the biological mechanisms causing IL-6 concentrations to be reduced in the TCA-graft treatment group were not caused or directly controlled by changes in the LCA profile. This also suggests that the changes in LCA concentrations in tissues and faeces were not dependent or directly controlled by the inflammatory profile or IL-6 concentrations in blood, although published studies have shown that diabetes development can increase LCA in tissues including the large intestine [20]. It is worth stating that the changes in LCA concentrations were not observed in all analysed tissues. LCA concentrations were reduced in plasma, large intestine, liver, spleen and faeces, while concentrations were not significantly different in the small intestine, compared with control. In some parts of the body, LCA was not detected, including brain, kidney, stomach, heart, skeletal muscle and white adipose tissue, regardless of TCA incorporation, which suggests that LCA accumulation is tissue specific. Hence, although TCA-graft treatment caused alteration in the biodistribution of the secondary bile acid, LCA, the effects were localised in particular tissues. These changes are consistent with published studies by other researchers. Studies in the literature have demonstrated an association between bile acids and diabetes treatment. Gu Y et al. investigated the relationship between diabetes treatment and the bile acid profile, in conjunction with the metabolic profile of gut microbiota. The authors showed that treatment with some antidiabetic drugs can elevate the ratio of primary to secondary bile acids as well as plasma concentrations of unconjugated bile acids in diabetic patients. Such effects of antidiabetic drugs were related to their effects on gut microbiota that naturally metabolise bile acids. The authors found that the antidiabetic drug Acarbose increased the relative abundances of Lactobacillus and Bifidobacterium in the gut microbiota and depletes Bacteroides, hence, altered bile acid metabolism and the bile acid profile. The authors conclude that the type of treatment and effects on gut microbiota can play a major role in diabetes outcome via modification of the bile acid profile [80]. Other studies in the literature have demonstrated a significant influence of bile acids in islet functions, glycaemic control and T1D treatment. Engin et al. examined the biological effects of the bile acid, tauroursodeoxycholic acid, in T1D development and treatment. The authors found that administration of tauroursodeoxycholic acid resulted in a significant reduction in diabetes development and improved prognosis [81]. Compared with our previous studies, LCA concentrations were shown to be modulated by diabetes development which suggests either direct or indirect effects on the bile acid pool [20]. Accordingly, our results showed that TCA incorporation to the transplanted graft influenced the bile acid profile, and influenced inflammation, although there was no clear and significant correlation between the changes in the bile acid profile and inflammation.

## 4. Conclusions

Since TCA direct effects on encapsulated primary islets’ functions in preclinical diabetic animals have not been investigated, particularly in relation to bile acid profile, this study has unique novelty. Our study aimed to explore the effects of TCA incorporation in islet-grafts, diabetes treatment, inflammation and the bile acid profile, in T1D. TCA incorporation into the graft showed better outcomes, compared with the control—possibly via anti-inflammatory effects, improved glycaemic control or reduction in the concentrations of the secondary bile acid, LCA. However, this is an acute study and thus the role of TCA might only be in supporting the encapsulated islets to survive and function in the short term. Future studies may explore the long-term effects of TCA through prolongation of the survival of the animals, possibly by insulin co-administration.

## Data Availability

The data presented in this study are available on request from the corresponding author. The data are not publicly available due to author property agreements.

## References

[1] Rokstad A.M., Lacik I., de Vos P., Strand B.L. (2013). Advances in biocompatibility and physico-chemical characterization of microspheres for cell encapsulation. Adv. Drug Deliv. Rev..

[2] Rydgren T., Sandler S. (2002). Efficacy of 1400 W, a novel inhibitor of inducible nitric oxide synthase, in preventing interleukin-1beta-induced suppression of pancreatic islet function in vitro and multiple low-dose streptozotocin-induced diabetes in vivo. Eur. J. Endocrinol..

[3] Allison J., McClive P., Oxbrow L., Baxter A., Morahan G., Miller J.F. (1994). Genetic requirements for acceleration of diabetes in non-obese diabetic mice expressing interleukin-2 in islet beta-cells. Eur. J. Immunol..

[4] Peakman M., Wen L., McNab G.L., Watkins P.J., Tan K.C., Vergani D. (1994). T cell clones generated from patients with type 1 diabetes using interleukin-2 proliferate to human islet antigens. Autoimmunity.

[5] Park Y.J., Warnock G.L., Ao Z., Safikhan N., Meloche M., Asadi A., Kieffer T.J., Marzban L. (2017). Dual role of interleukin-1β in islet amyloid formation and its β-cell toxicity: Implications for type 2 diabetes and islet transplantation. Diabetes Obes. Metab..

[6] Rabinovitch A., Suarez-Pinzon W.L., Sorensen O., Bleackley R.C., Power R.F., Rajotte R.V. (1995). Combined therapy with interleukin-4 and interleukin-10 inhibits autoimmune diabetes recurrence in syngeneic islet-transplanted nonobese diabetic mice. Analysis of cytokine mRNA expression in the graft. Transplantation.

[7] Ladefoged M., Buschard K., Hansen A.M. (2013). Increased expression of toll-like receptor 4 and inflammatory cytokines, interleukin-6 in particular, in islets from a mouse model of obesity and type 2 diabetes. APMIS.

[8] Arif S., Moore F., Marks K., Bouckenooghe T., Dayan C.M., Planas R., Vives-Pi M., Powrie J., Tree T., Marchetti P. (2011). Peripheral and islet interleukin-17 pathway activation characterizes human autoimmune diabetes and promotes cytokine-mediated beta-cell death. Diabetes.

[9] Kaas A., Pfleger C., Hansen L., Buschard K., Schloot N.C., Roep B.O., Mortensen H.B., Hvidore Study Group on Childhood D. (2010). Association of adiponectin, interleukin (IL)-1ra, inducible protein 10, IL-6 and number of islet autoantibodies with progression patterns of type 1 diabetes the first year after diagnosis. Clin. Exp. Immunol..

[10] Krishnan R., Alexander M., Robles L., Foster C.E., Lakey J.R. (2014). Islet and stem cell encapsulation for clinical transplantation. Rev. Diabet Stud..

[11] Gedawy A., Al-Salami H., Dass C.R. (2020). Advanced and multifaceted stability profiling of the first-line antidiabetic drugs metformin, gliclazide and glipizide under various controlled stress conditions. Saudi Pharm. J..

[12] Mooranian A., Negrulj R., Takechi R., Jamieson E., Morahan G., Al-Salami H. (2017). Alginate-combined cholic acid increased insulin secretion of microencapsulated mouse cloned pancreatic beta cells. Ther. Deliv..

[13] Suciu M., Ionescu C.M., Ciorita A., Tripon S.C., Nica D., Al-Salami H., Barbu-Tudoran L. (2020). Applications of superparamagnetic iron oxide nanoparticles in drug and therapeutic delivery, and biotechnological advancements. Beilstein J. Nanotechnol..

[14] Al-Salami H., Kansara H., King J., Morar B., Jayathilaka B., Fawcett P.J., Mikov M. (2007). Bile acids: A bitter sweet remedy for diabetes. New Zealand Pharm. J..

[15] Wong C.Y., Al-Salami H., Dass C.R. (2020). Current status and applications of animal models in pre-clinical development of orally administered insulin-loaded nanoparticles. J. Drug Target..

[16] Mooranian A., Zamani N., Takechi R., Luna G., Mikov M., Golocorbin-Kon S., Elnashar M., Arfuso F., Al-Salami H. (2019). An in vivo pharmacological study: Variation in tissue-accumulation for the drug probucol as the result of targeted microtechnology and matrix-acrylic acid optimization and stabilization techniques. PLoS ONE.

[17] Wagle S.R., Walker D., Kovacevic B., Gedawy A., Mikov M., Golocorbin-Kon S., Mooranian A., Al-Salami H. (2020). Micro-Nano formulation of bile-gut delivery: Rheological, stability and cell survival, basal and maximum respiration studies. Sci. Rep..

[18] Wagle S.R., Kovacevic B., Walker D., Ionescu C.M., Shah U., Stojanovic G., Kojic S., Mooranian A., Al-Salami H. (2020). Alginate-based drug oral targeting using bio-micro/nano encapsulation technologies. Expert Opin. Drug Deliv..

[19] Wagle S.R., Kovacevic B., Walker D., Ionescu C.M., Jones M., Stojanovic G., Kojic S., Mooranian A., Al-Salami H. (2020). Pharmacological and advanced cell respiration effects, enhanced by toxic human-bile nano-pharmaceuticals of probucol cell-targeting formulations. Pharmaceutics.

[20] Mooranian A., Zamani N., Takechi R., Luna G., Mikov M., Goločorbin-Kon S., Kovacevic B., Arfuso F., Al-Salami H. (2020). Modulatory nano/micro effects of diabetes development on pharmacology of primary and secondary bile acids concentrations. Curr. Diabetes Rev..

[21] Mooranian A., Zamani N., Mikov M., Goločorbin-Kon S., Stojanovic G., Arfuso F., Kovacevic B., Al-Salami H. (2020). A second-generation micro/nano capsules of an endogenous primary unmetabolised bile acid, stabilized by Eudragit-alginate complex with antioxidant compounds. Saudi Pharm. J..

[22] Mooranian A., Zamani N., Mikov M., Goločorbin-Kon S., Stojanovic G., Arfuso F., Kovacevic B., Al-Salami H. (2020). Bio micro-nano technologies of antioxidants optimised their pharmacological and cellular effects, ex vivo, in pancreatic β-cells. Nanotechnol. Sci. Appl..

[23] Mooranian A., Zamani N., Kovacevic B., Ionescu C.M., Luna G., Mikov M., Goločorbin-Kon S., Stojanovic G., Kojic S., Al-Salami H. (2020). Pharmacological effects of secondary bile acid microparticles in diabetic murine model. Curr. Diabetes Rev..

[24] Mooranian A., Zamani N., Ionescu C.M., Takechi R., Luna G., Mikov M., Goločorbin-Kon S., Kovačević B., Al-Salami H. (2020). Oral gavage of nano-encapsulated conjugated acrylic acid-bile acid formulation in type 1 diabetes altered pharmacological profile of bile acids, and improved glycaemia and suppressed inflammation. Pharmacol. Rep..

[25] Mooranian A., Raj Wagle S., Kovacevic B., Takechi R., Mamo J., Lam V., Watts G.F., Mikov M., Golocorbin-Kon S., Stojanovic G. (2020). Bile acid bio-nanoencapsulation improved drug targeted-delivery and pharmacological effects via cellular flux: 6-months diabetes preclinical study. Sci. Rep..

[26] Mathavan S., Ionescu C.M., Kovacevic B., Mikov M., Golocorbin-Kon S., Mooranian A., Dass C.R., Al-Salami H. (2020). Histological effects of pharmacologically active human bile acid nano/micro-particles in Type-1 diabetes. Ther. Deliv..

[27] Jones M., Walker D., Ionescu C.M., Kovacevic B., Wagle S.R., Mooranian A., Brown D., Al-Salami H. (2020). Microencapsulation of Coenzyme Q10 and bile acids using ionic gelation vibrational jet flow technology for oral delivery. Ther. Deliv..

[28] Mooranian A., Zamani N., Takechi R., Al-Sallami H., Mikov M., Goločorbin-Kon S., Kovacevic B., Arfuso F., Al-Salami H. (2019). Probucol-poly(meth)acrylate-bile acid nanoparticles increase IL-10, and primary bile acids in prediabetic mice. Ther. Deliv..

[29] Mooranian A., Zamani N., Mikov M., Goločorbin-Kon S., Stojanovic G., Arfuso F., Al-Salami H. (2019). Stability and biological testing of taurine-conjugated bile acid antioxidant microcapsules for diabetes treatment. Ther. Deliv..

[30] Mooranian A., Zamani N., Luna G., Al-Sallami H., Mikov M., Goločorbin-Kon S., Stojanovic G., Arfuso F., Kovacevic B., Al-Salami H. (2019). Bile acid-polymer-probucol microparticles: Protective effect on pancreatic β-cells and decrease in type 1 diabetes development in a murine model. Pharm. Dev. Technol..

[31] Mathavan S., Chen-Tan N., Arfuso F., Al-Salami H. (2016). The role of the bile acid chenodeoxycholic acid in the targeted oral delivery of the anti-diabetic drug gliclazide, and its applications in type 1 diabetes. Artif. Cells Nanomed. Biotechnol..

[32] Mooranian A., Negrulj R., Arfuso F., Al-Salami H. (2016). Multicompartmental, multilayered probucol microcapsules for diabetes mellitus: Formulation characterization and effects on production of insulin and inflammation in a pancreatic β-cell line. Artif. Cells Nanomed. Biotechnol..

[33] Mooranian A., Negrulj R., Arfuso F., Al-Salami H. (2016). Characterization of a novel bile acid-based delivery platform for microencapsulated pancreatic beta-cells. Artif. Cells Nanomed. Biotechnol..

[34] Mooranian A., Negrulj R., Chen-Tan N., Fakhoury M., Arfuso F., Jones F., Al-Salami H. (2016). Advanced bile acid-based multi-compartmental microencapsulated pancreatic beta-cells integrating a polyelectrolyte-bile acid formulation, for diabetes treatment. Artif. Cells Nanomed. Biotechnol..

[35] Mooranian A., Negrulj R., Jamieson E., Morahan G., Al-Salami H. (2016). Biological Assessments of Encapsulated Pancreatic β-Cells: Their Potential Transplantation in Diabetes. Cell. Mol. Bioeng..

[36] Mathavan S., Ionescu C.M., Kovacevic B., Mikov M., Golocorbin-Kon S., Mooranian A., Dass C.R., Al-Salami H. (2019). Formulation buoyancy of nanoencapsulated gliclazide using primary, conjugated and deconjugated bile acids. Ther. Deliv..

[37] Mamo J.C.L., Lam V., Brook E., Mooranian A., Al-Salami H., Fimognari N., Nesbit M., Takechi R. (2019). Probucol prevents blood–brain barrier dysfunction and cognitive decline in mice maintained on pro-diabetic diet. Diabetes Vasc. Dis. Res..

[38] Mooranian A., Zamani N., Takechi R., Al-Sallami H., Mikov M., Goločorbin-Kon S., Kovacevic B., Arfuso F., Al-Salami H. (2018). Pharmacological effects of nanoencapsulation of human-based dosing of probucol on ratio of secondary to primary bile acids in gut, during induction and progression of type 1 diabetes. Artif. Cells Nanomed. Biotechnol..

[39] Mooranian A., Zamani N., Mikov M., Goločorbin-Kon S., Stojanovic G., Arfuso F., Al-Salami H. (2018). Novel nano-encapsulation of probucol in microgels: Scanning electron micrograph characterizations, buoyancy profiling, and antioxidant assay analyses. Artif. Cells Nanomed. Biotechnol..

[40] Mooranian A., Zamani N., Mikov M., Goločorbin-Kon S., Stojanovic G., Arfuso F., Al-Salami H. (2018). Eudragit^®^-based microcapsules of probucol with a gut-bacterial processed secondary bile acid. Ther. Deliv..

[41] Mooranian A., Takechi R., Jamieson E., Morahan G., Al-Salami H. (2018). The effect of molecular weights of microencapsulating polymers on viability of mouse-cloned pancreatic Î²-cells: Biomaterials, osmotic forces and potential applications in diabetes treatment. Pharm. Dev. Technol..

[42] Mooranian A., Negrulj R., Takechi R., Mamo J., Al-Sallami H., Al-Salami H. (2018). The biological effects of the hypolipidaemic drug probucol microcapsules fed daily for 4 weeks, to an insulin-resistant mouse model: Potential hypoglycaemic and anti-inflammatory effects. Drug Deliv. Transl. Res..

[43] Mooranian A., Negrulj R., Takechi R., Jamieson E., Morahan G., Al-Salami H. (2018). Influence of Biotechnological Processes, Speed of Formulation Flow and Cellular Concurrent Stream-Integration on Insulin Production from β-cells as a Result of Co-Encapsulation with a Highly Lipophilic Bile Acid. Cell. Mol. Bioeng..

[44] Mooranian A., Negrulj R., Takechi R., Jamieson E., Morahan G., Al-Salami H. (2018). Electrokinetic potential-stabilization by bile acid-microencapsulating formulation of pancreatic β-cells cultured in high ratio poly-L-ornithine-gel hydrogel colloidal dispersion: Applications in cell-biomaterials, tissue engineering and biotechnological applications. Artif. Cells Nanomed. Biotechnol..

[45] Mamo J.C., Lam V., Al-Salami H., Brook E., Mooranian A., Nesbit M., Graneri L., D’Alonzo Z., Fimognari N., Stephenson A. (2018). Sodium alginate capsulation increased brain delivery of probucol and suppressed neuroinflammation and neurodegeneration. Ther. Deliv..

[46] Takechi R., Lam V., Brook E., Giles C., Fimognari N., Mooranian A., Al-Salami H., Coulson S.H., Nesbit M., Mamo J.C.L. (2017). Blood-brain barrier dysfunction precedes cognitive decline and neurodegeneration in diabetic insulin resistant mouse model: An implication for causal link. Front. Aging Neurosci..

[47] Mooranian A., Tackechi R., Jamieson E., Morahan G., Al-Salami H. (2017). Innovative Microcapsules for Pancreatic β-Cells Harvested from Mature Double-Transgenic Mice: Cell Imaging, Viability, Induced Glucose-Stimulated Insulin Measurements and Proinflammatory Cytokines Analysis. Pharm. Res..

[48] Mooranian A., Negrulj R., Takechi R., Jamieson E., Morahan G., Al-Salami H. (2017). New Biotechnological Microencapsulating Methodology Utilizing Individualized Gradient-Screened Jet Laminar Flow Techniques for Pancreatic β-Cell Delivery: Bile Acids Support Cell Energy-Generating Mechanisms. Mol. Pharm..

[49] Mooranian A., Negrulj R., Al-Salami H. (2017). The effects of Ionic Gelation- Vibrational Jet Flow technique in fabrication of microcapsules incorporating β-cell: Applications in Type-1 Diabetes. Curr. Diabetes Rev..

[50] Mamo J.C.L., Lam V., Giles C., Coulson S.H., Fimognari N., Mooranian A., Al-Salami H., Takechi R. (2017). Antihypertensive agents do not prevent blood-brain barrier dysfunction and cognitive deficits in dietary-induced obese mice. Int. J. Obes..

[51] Al-Salami H., Mamo J.C., Mooranian A., Negrulj R., Lam V., Elahy M., Takechi R. (2017). Long-Term Supplementation of Microencapsulated ursodeoxycholic Acid Prevents Hypertension in a Mouse Model of Insulin Resistance. Exp. Clin. Endocrinol. Diabetes.

[52] Negrulj R., Mooranian A., Chen-Tan N., Al-Salami H.S., Mikov M., Golocorbin-Kon S., Fakhoury M., Watts G.F., Arfuso F., Al-Salami H. (2016). Swelling, mechanical strength, and release properties of probucol microcapsules with and without a bile acid, and their potential oral delivery in diabetes. Artif. Cells Nanomed. Biotechnol..

[53] Mooranian A., Negrulj R., Al-Salami H. (2016). Viability and topographical analysis of microencapsulated β-cells exposed to a biotransformed tertiary bile acid: An ex vivo study. Int. J. Nano Biomater..

[54] Mooranian A., Negrulj R., Al-Salami H., Morahan G., Jamieson E. (2016). Designing anti-diabetic β-cells microcapsules using polystyrenic sulfonate, polyallylamine, and a tertiary bile acid: Morphology, bioenergetics, and cytokine analysis. Biotechnol. Prog..

[55] Mooranian A., Negrulj R., Al-Salami H. (2016). The incorporation of water-soluble gel matrix into bile acid-based microcapsules for the delivery of viable β-cells of the pancreas, in diabetes treatment: Biocompatibility and functionality studies. Drug Deliv. Transl. Res..

[56] Mooranian A., Negrulj R., Al-Salami H. (2016). Flow vibration-doubled concentric system coupled with low ratio amine to produce bile acid-macrocapsules of β-cells. Ther. Deliv..

[57] Mooranian A., Negrulj R., Al-Salami H. (2016). The impact of allylamine-bile acid combinations on cell delivery microcapsules in diabetes. J. Microencapsul..

[58] Mooranian A., Negrulj R., Al-Salami H. (2016). The Influence of Stabilized Deconjugated Ursodeoxycholic Acid on Polymer-Hydrogel System of Transplantable NIT-1 Cells. Pharm. Res..

[59] Gvoic M., Vukmirovic S., Al-Salami H., Mooranian A., Mikov M., Stankov K. (2021). Bile acids as novel enhancers of CNS targeting antitumor drugs: A comprehensive review. Pharm. Dev. Technol..

[60] Miller A.L., Leach M.C. (2015). Using the mouse grimace scale to assess pain associated with routine ear notching and the effect of analgesia in laboratory mice. Lab. Anim..

[61] Leach M.C., Klaus K., Miller A.L., Scotto di Perrotolo M., Sotocinal S.G., Flecknell P.A. (2012). The assessment of post-vasectomy pain in mice using behaviour and the Mouse Grimace Scale. PLoS ONE.

[62] Matsumiya L.C., Sorge R.E., Sotocinal S.G., Tabaka J.M., Wieskopf J.S., Zaloum A., King O.D., Mogil J.S. (2012). Using the Mouse Grimace Scale to reevaluate the efficacy of postoperative analgesics in laboratory mice. J. Am. Assoc. Lab. Anim. Sci..

[63] Mathavan S., Chen-Tan N., Arfuso F., Al-Salami H. (2018). Morphological, Stability, and Hypoglycemic Effects of New Gliclazide-Bile Acid Microcapsules for Type 1 Diabetes Treatment: The Microencapsulation of Anti-diabetics Using a Microcapsule-Stabilizing Bile Acid. AAPS PharmSciTech.

[64] Wagle S.R., Kovacevic B., Ionescu C.M., Walker D., Jones M., Carey L., Takechi R., Mikov M., Mooranian A., Al-Salami H. (2021). pharmacological and Biological Study of Microencapsulated Probucol-Secondary Bile Acid in a Diseased Mouse Model. Pharmaceutics.

[65] Al-Salami H., Butt G., Tucker I., Mikov M. (2008). Influence of the semisynthetic bile acid (MKC) on the ileal permeation of gliclazide in healthy and diabetic rats. Pharmacol. Rep..

[66] Mikov M., Boni N.S., Al-Salami H., Kuhajda K., Kevresan S., Golocorbin-Kon S., Fawcett J.P. (2007). Bioavailability and hypoglycemic activity of the semisynthetic bile acid salt, sodium 3alpha,7alpha-dihydroxy-12-oxo-5beta-cholanate, in healthy and diabetic rats. Eur. J. Drug Metab. Pharm..

[67] de Vos P., Faas M.M., Strand B., Calafiore R. (2006). Alginate-based microcapsules for immunoisolation of pancreatic islets. Biomaterials.

[68] De Vos P., De Haan B.J., Wolters G.H., Strubbe J.H., Van Schilfgaarde R. (1997). Improved biocompatibility but limited graft survival after purification of alginate for microencapsulation of pancreatic islets. Diabetologia.

[69] Mooraniana A., Negrulja R., Chen-Tanb N., Al-Sallamic H.S., Fangd Z., Mikov M., Golocorbin-Kong S., Fakhouri M., Arfusoe F., Al-Salamia H. (2014). Novel artificial cell microencapsulation of a complex gliclazide-deoxycholic bile acid formulation: A Characterisation Study. Drug Des. Dev. Ther..

[70] Al-Salami H., Butt G., Fawcett J.P., Tucker I.G., Golocorbin-Kon S., Mikov M. (2008). Probiotic treatment reduces blood glucose levels and increases systemic absorption of gliclazide in diabetic rats. Eur. J. Drug Metab. Pharm..

[71] Al-Salami H., Butt G., Tucker I., Fawcett P.J., Golocorbin-Kon S., Mikov I., Mikov M. (2009). Gliclazide reduces MKC intestinal transport in healthy but not diabetic rats. Eur. J. Drug Metab. Pharmacokinet..

[72] Mooranian A., Negrulj R., Al-Salami H. (2016). Alginate-deoxycholic Acid Interaction and Its Impact on Pancreatic Β-Cells and Insulin Secretion and Potential Treatment of Type 1 Diabetes. J. Pharm. Innov..

[73] Jin S.M., Oh S.H., Oh B.J., Suh S., Bae J.C., Lee J.H., Lee M.S., Lee M.K., Kim K.W., Kim J.H. (2014). Benefits of PEGylation in the early post-transplant period of intraportal islet transplantation as assessed by magnetic resonance imaging of labeled islets. Islets.

[74] Loganathan G., Graham M.L., Radosevich D.M., Soltani S.M., Tiwari M., Anazawa T., Papas K.K., Sutherland D.E., Hering B.J., Balamurugan A.N. (2013). Factors affecting transplant outcomes in diabetic nude mice receiving human, porcine, and nonhuman primate islets: Analysis of 335 transplantations. Transplantation.

[75] Stokes R.A., Cheng K., Deters N., Lau S.M., Hawthorne W.J., O’Connell P.J., Stolp J., Grey S., Loudovaris T., Kay T.W. (2013). Hypoxia-inducible factor-1alpha (HIF-1alpha) potentiates beta-cell survival after islet transplantation of human and mouse islets. Cell Transpl..

[76] Chen C., Moreno R., Samikannu B., Bretzel R.G., Schmitz M.L., Linn T. (2011). Improved intraportal islet transplantation outcome by systemic IKK-beta inhibition: NF-kappaB activity in pancreatic islets depends on oxygen availability. Am. J. Transpl..

[77] Schneider S., Feilen P.J., Brunnenmeier F., Minnemann T., Zimmermann H., Zimmermann U., Weber M.M. (2005). Long-term graft function of adult rat and human islets encapsulated in novel alginate-based microcapsules after transplantation in immunocompetent diabetic mice. Diabetes.

[78] Wang T., Adcock J., Kuhtreiber W., Qiang D., Salleng K.J., Trenary I., Williams P. (2008). Successful allotransplantation of encapsulated islets in pancreatectomized canines for diabetic management without the use of immunosuppression. Transplantation.

[79] Beger C., Cirulli V., Vajkoczy P., Halban P.A., Menger M.D. (1998). Vascularization of purified pancreatic islet-like cell aggregates (pseudoislets) after syngeneic transplantation. Diabetes.

[80] Gu Y., Wang X., Li J., Zhang Y., Zhong H., Liu R., Zhang D., Feng Q., Xie X., Hong J. (2017). Analyses of gut microbiota and plasma bile acids enable stratification of patients for antidiabetic treatment. Nat. Commun..

[81] Engin F., Yermalovich A., Nguyen T., Hummasti S., Fu W., Eizirik D.L., Mathis D., Hotamisligil G.S. (2013). Restoration of the unfolded protein response in pancreatic beta cells protects mice against type 1 diabetes. Sci. Transl. Med..

